# MASLD in type 2 diabetes: steatosis–fibrosis burden and metabolic determinants in a real-world cohort

**DOI:** 10.1007/s00592-026-02791-5

**Published:** 2026-09-10

**Authors:** Salvatore Corrao, Salvatore Scibetta, Brenda Bongiorno, Carola Lopriore, Luigi Calvo

**Affiliations:** 1 Department of Internal Medicine, National Relevance and High Specialisation Hospital Trust ARNAS Civico Di Cristina Benfratelli, Palermo, 90127 Italy; 2 Institute for Biomedical Research and Innovation, National Research Council (IRIB-CNR), Palermo, Italy; 3 Department of Health Promotion Sciences, Maternal and Infant Care, Internal Medicine and Medical Specialities (PROMISE), University of Palermo, Palermo, 90127 Italy; 4 Department of Clinical Medicine and Head of Internal Medicine Unit, National Relevance and High Specialisation Hospital Trust ARNAS Ospedale Civico, P.zza Nicola Leotta, 4, Palermo, 90127 Italy

**Keywords:** MASLD, Type 2 diabetes, Central obesity, Sex differences, Liver fibrosis, Shear-wave elastography

## Abstract

**Aims:**

Metabolic dysfunction-associated steatotic liver disease (MASLD) is highly prevalent in type 2 diabetes mellitus (T2DM), but the burden of steatosis and fibrosis and their associated factors remain incompletely defined. We assessed the prevalence, severity, and factors associated with of MASLD in a real-world cohort of patients with T2DM.

**Methods:**

In this cross-sectional study, 219 consecutive outpatients with T2DM underwent anthropometric laboratory, ultrasonographic and shear-wave elastographic assessment. MASLD was defined by the multisociety Delphi consensus. Independent associations were evaluated using multivariable logistic regression, with waist circumference as the primary adiposity measure.

**Results:**

MASLD was diagnosed in 75.3% of participants. Severe steatosis (S3) was present in 11.9%, clinically significant fibrosis (F ≥ 2) in 7.3%, and advanced fibrosis (F ≥ 3) in 5.5%. Male sex (OR 2.49, 95% CI 1.26–4.94) and waist circumference (OR 1.04 per cm, 95% CI 1.01–1.07) were independently associated with MASLD. Severe steatosis was independently associated with male sex and waist circumference. MASLD prevalence increased across waist circumference quartiles (trend *p* = 0.001), while the adjusted odds plateaued in the upper half, paralleled by a higher prevalence of severe steatosis (*p* < 0.001), whereas fibrosis was not associated with abdominal adiposity (*p* = 0.65). HbA1c and diabetes duration did not differ between groups in univariable comparisons, and age was not associated with MASLD in the multivariable models.

**Conclusions:**

MASLD is highly prevalent in T2DM and is independently associated with male sex and central adiposity. Abdominal adiposity identifies steatosis risk in a graded manner but does not discriminate fibrosis, supporting adiposity- and sex-informed hepatic phenotyping in patients with T2DM.

**Supplementary Information:**

The online version contains supplementary material available at https://doi.org/10.1007/s00592-026-02791-5.

## Introduction

Metabolic dysfunction-associated steatotic liver disease (MASLD) represents the current conceptual framework for hepatic steatosis arising in the context of cardiometabolic dysfunction. Endorsed by a multisociety Delphi consensus, the term replaced “non-alcoholic fatty liver disease” (NAFLD) and requires hepatic steatosis together with at least one cardiometabolic risk factor, thereby anchoring the diagnosis to the underlying metabolic substrate rather than to the exclusion of alternative aetiologies [1]. This nomenclature followed an earlier consensus that had proposed the intermediate term MAFLD [2]. More than 95% of patients formerly classified as NAFLD satisfy the revised MASLD criteria, and the reclassification has reframed steatotic liver disease as a manifestation of systemic metabolic dysfunction [1, 3].

Within this framework, type 2 diabetes mellitus (T2DM) occupies a position of particular relevance. Steatosis is not only highly prevalent in T2DM but is also the soil from which clinically meaningful disease develops: it is the progression to fibrosis, rather than steatosis itself, that drives liver-related morbidity and long-term cardiovascular risk [4]. A recent meta-analysis of more than two million patients estimated the pooled prevalence of MASLD in T2DM at approximately 65% [5], and current guidance increasingly recommends active case-finding for advanced fibrosis in people with diabetes [6–8]. Despite this, the magnitude and severity of the hepatic burden in consecutively enrolled, real-world diabetic populations remains incompletely characterised, and most prevalence estimates derive from mixed metabolic cohorts in which diabetes is one of several contributing factors [9].

This distinction is methodologically important. When diabetes is treated as a variable in a heterogeneous population, it inevitably emerges as a dominant correlate of MASLD, and its weight may obscure the contribution of other factors. A complementary and clinically actionable question is what continues to discriminate MASLD once diabetes is universal—that is, among patients who already carry the prevailing cardiometabolic risk factor. If sex, central obesity and adiposity retain their discriminating value in this setting, risk stratification for the diabetic liver cannot stop at the diagnosis of diabetes.

Sex differences add a further layer. Men exhibit a higher prevalence and severity of steatotic liver disease during reproductive age, a difference attributed to a more visceral pattern of fat distribution and to sex-hormone influences on hepatic lipid metabolism [10, 11]; after menopause, women approach and may exceed the risk observed in men as oestrogenic protection is lost [12, 13]. These influences are expected to be especially pronounced in a diabetic cohort enriched for central adiposity.

Against this background, we conducted a cross-sectional study in a real-world cohort of outpatients with T2DM. We first quantified the hepatic burden—the prevalence of MASLD, the distribution of steatosis severity, and the prevalence of fibrosis assessed by shear-wave elastography—and then examined the independent metabolic factors associated with MASLD in a population in which diabetes is held constant. As a secondary, hypothesis-generating analysis, and with full acknowledgement of the limitations of a cross-sectional design, we explored the relationship between glucose-lowering therapy classes and the hepatic phenotype.

## Methods

### Study design and population

This single-centre cross-sectional study consecutively enrolled eligible outpatients with type 2 diabetes mellitus attending the Internal Medicine outpatient clinic of ARNAS Civico Di Cristina Benfratelli, Palermo, Italy. All participants had an established diagnosis of T2DM and at least one additional component of the metabolic syndrome. Patients were recruited consecutively between May 2024 and October 2025; the present cohort does not overlap with the population of our previous study [9]. Anthropometric measurements (height, weight, body mass index [BMI] and waist circumference), blood pressure and laboratory variables were recorded, together with current glucose-lowering and lipid-lowering therapy and diabetes duration. Missing laboratory data reflected routine clinical practice and were not imputed because of the exploratory nature of the study. The study was conducted using data from the departmental clinical research registry. All participants provided written informed consent. The study was approved by the Ethics Committee Comitato Etico Locale Palermo 2 (Approval No. 132 CIVICO 2025; 20 November 2025).

### Hepatic steatosis and visceral fat

Abdominal ultrasonography was performed on an Esaote MyLab X8 PRO ultrasound system (Esaote, Genoa, Italy) using a convex probe. Steatosis was assessed by B-mode as the reference first-line technique [14]. Increased hepatic echogenicity relative to the renal cortex (“bright liver”), together with posterior beam attenuation and blurring of the intrahepatic vessel walls, was used as the qualitative marker of steatosis and graded as mild (S1), moderate (S2) or severe (S3); diaphragmatic attenuation was used to distinguish moderate from severe involvement, and the assessment is qualitative and operator-dependent. Visceral fat thickness was measured with the same convex probe ultrasonographically as the distance, in millimetres, from the inner edge of the posterior rectus abdominis muscle to the anterior wall of the aorta [15].

### Shear-wave elastography

Liver stiffness was measured by two-dimensional shear-wave elastography (2D-SWE) using the integrated QElaXto module of the same Esaote MyLab X8 PRO system with the convex probe, which quantifies tissue elasticity from the propagation speed of focused-ultrasound-induced shear waves, with stiffer tissue yielding faster waves [16]. Examinations were performed after at least six hours’ fasting, with the patient supine and the right arm in maximal abduction, through an intercostal approach over the right hepatic lobe; the region of interest was positioned at least 1.5–2 cm below the liver capsule, avoiding large vessels, bile ducts and rib shadowing, during a short breath-hold in neutral respiration. For each patient the median of at least five valid acquisitions was recorded and expressed as Young’s modulus in kilopascals (kPa); acquisitions were considered reliable when the interquartile-range/median ratio was ≤ 30%. Steatosis grading and 2D-SWE were performed independently by two experienced operators who were blinded to laboratory data; inter-operator agreement was assessed in cases of discordance and was excellent (Cohen’s κ > 0.90), with residual disagreements resolved by consensus. Because elastographic cut-offs are not interchangeable across systems, the following categories, suggested by 2D-SWE, were applied [17, 18]: ≤5.3 kPa (no fibrosis, F0); 5.31–6.99 kPa (F1); 7.0–8.9 kPa (F2); 9.0–11.9 kPa (F3); and ≥ 12 kPa (F4, cirrhosis).

### Diagnostic criteria

MASLD was diagnosed according to the Delphi consensus: ultrasonographic hepatic steatosis together with at least one of five cardiometabolic risk factors (BMI ≥ 25 kg/m² or waist circumference > 94 cm in men / >80 cm in women; fasting glucose ≥ 5.6 mmol/L, HbA1c ≥ 5.7% (≥ 39 mmol/mol) or treated T2DM; blood pressure ≥ 130/85 mmHg or treatment; triglycerides ≥ 1.70 mmol/L or treatment; HDL cholesterol ≤ 1.0 mmol/L in men / ≤1.3 mmol/L in women or treatment) [1]. Alcohol intake and coexisting liver diseases (viral hepatitis and steatogenic medications) were ascertained from the clinical registry and the multisociety steatotic-liver-disease classification was applied (MASLD thresholds < 140 g alcohol/week in women and < 210 g/week in men; MetALD 140–350 and 210–420 g/week, respectively; ALD above these ranges). Central obesity was defined according to both IDF [19] and NCEP-ATP III [20] criteria, and the metabolic syndrome according to NCEP-ATP III [20].

### Statistical analysis

Categorical variables are presented as counts and percentages and continuous variables as median (interquartile range, Q1–Q3). Between-group comparisons used the Mann–Whitney U test for continuous variables and the χ² test or Fisher’s exact test, as appropriate, for proportions. The independent association between MASLD and candidate factors was assessed by multivariable logistic regression; odds ratios (OR) with 95% confidence intervals (CI) are reported. Adiposity was modelled using waist circumference as the primary continuous measure; because waist circumference and BMI were collinear, they were not entered jointly, and two pre-specified MASLD models were fitted, each including sex and age together with one adiposity measure (waist circumference, Model A; BMI, Model B). Severe steatosis (S3) was modelled with sex, age and waist circumference. The relationship between abdominal adiposity and the hepatic phenotype was further characterised by dividing waist circumference into quartiles and estimating, for each quartile, the prevalence of MASLD, severe steatosis and fibrosis; trends across quartiles were tested with the Cochran–Armitage test, and quartile-specific odds ratios for MASLD (lowest quartile as reference) were obtained from a logistic model adjusted for sex and age. Effect modification by sex was assessed by adding a sex-by-waist interaction term, and sex-specific probabilities of MASLD were estimated across the observed range of waist circumference from a model including sex, age and waist circumference. Where complete or quasi-complete separation precluded stable maximum-likelihood estimation, Firth’s penalised-likelihood logistic regression was used [21] The association between glucose-lowering and lipid-lowering therapy classes and the hepatic outcomes (MASLD, severe steatosis and prevalent fibrosis) was examined as a secondary, exploratory analysis in separate models, each adjusted for age, sex and waist circumference, with Firth’s method applied to the sparse outcomes; the dipeptidyl peptidase-4 inhibitor class was excluded owing to its small number of users (*n* = 3). No adjustment for multiplicity was applied across the 18 exploratory comparisons. Variables with substantial missingness were retained for descriptive purposes but excluded from regression models. A two-tailed *p* < 0.05 was considered significant. All analyses were performed in Stata 19 (StataCorp LLC, College Station, TX); Firth’s penalised-likelihood logistic regression was implemented with the firthlogit command.

## Results

### Cohort characteristics

**Fig. 1:**
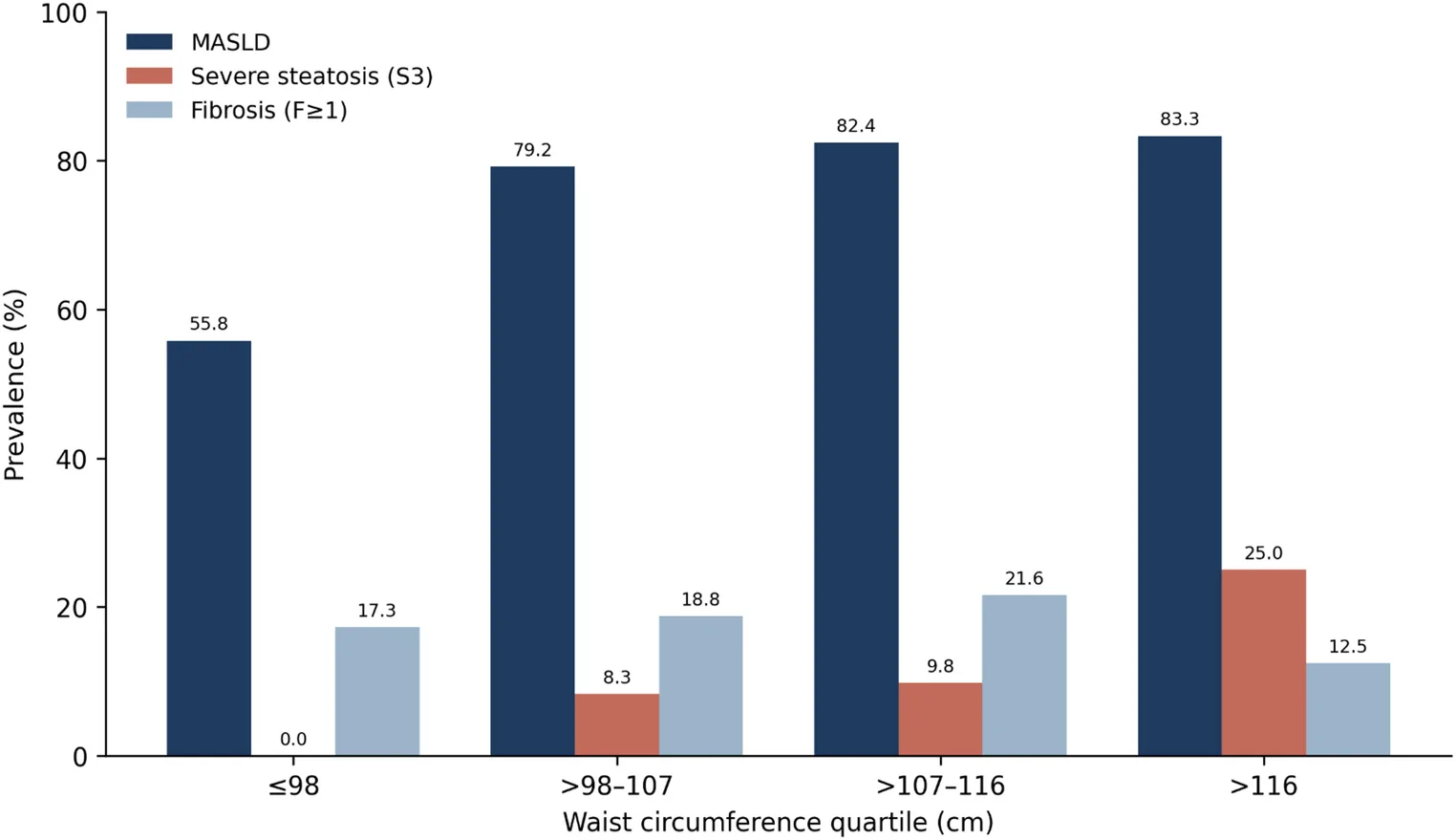
Dissociation of steatosis and fibrosis across the spectrum of abdominal adiposity. Prevalence of MASLD, severe steatosis (S3) and fibrosis (F ≥ 1) by quartile of waist circumference (Q1 ≤ 98, Q2 > 98–107, Q3 > 107–116, Q4 > 116 cm; *N* = 199). The prevalence of MASLD (55.8%→83.3%) and of severe steatosis (0%→25.0%) rises progressively with abdominal adiposity (Cochran–Armitage test for trend *p* = 0.001 and *p* < 0.001, respectively), whereas fibrosis shows no corresponding gradient (17.3%→12.5%; *p* = 0.65). The contrast indicates that abdominal adiposity is coupled to hepatic fat accumulation but not, in this cohort, to fibrogenesis, and that anthropometric markers should not be used as surrogates for fibrosis risk

A total of 219 outpatients with type 2 diabetes were enrolled. The median age was 65 years (IQR 57.5–73.0) and 57.5% were men. Adiposity was prominent: median BMI was 29.7 kg/m² (26.4–33.4) and median waist circumference 107.3 cm (98.0–116.0), with central obesity present in 92.0% of patients by IDF and 78.4% by NCEP-ATP III criteria, and median visceral fat thickness of 66.0 mm. The cohort was metabolically loaded, with hypertension in 78.5%, dyslipidaemia in 84.9%, the metabolic syndrome in 53.4% and coronary artery disease in 23.3%. Median diabetes duration was 10 years and median HbA1c 6.6% (49 mmol/mol). Glucose-lowering treatment comprised metformin in 58.0%, GLP1-RA in 52.5%, SGLT2i in 32.6% and insulin in 15.6%; 70.8% were on statins. Waist circumference was available in 199 of 219 participants (90.9%); waist-based percentages, the quartile analysis (Sect.  3.4; Table 4; Fig. 1) and the waist-adjusted models refer to this subgroup. Full baseline characteristics are reported in Table 1.

### Hepatic burden

MASLD was present in 165 patients (75.3%). Ultrasonographic steatosis of any grade was detected in 75.3%, distributed as mild (S1) in 30.1%, moderate (S2) in 33.3% and severe (S3) in 11.9%; because every participant had T2DM and no case met MetALD, ALD or cryptogenic-SLD criteria, all patients with steatosis were classified as MASLD. By shear-wave elastography, median liver stiffness was 4.4 kPa (3.8–5.1) and most patients had no measurable fibrosis (F0, 82.6%). Fibrosis of any stage (F ≥ 1) was present in 17.4%, but clinically relevant fibrosis (F ≥ 2) was confined to 7.3% and advanced fibrosis (F ≥ 3) to 5.5% (F1 10.0%, F2 1.8%, F3 2.7%, F4 2.7%). Thus, in this real-world diabetic cohort the steatotic burden was high and frequently severe, whereas the fibrotic burden, although clinically meaningful in a minority, remained comparatively limited.

### Factors associated with MASLD

In univariate comparison (Table 2), MASLD patients had significantly higher BMI (30.1 vs. 27.8 kg/m², *p* = 0.009), waist circumference (109.0 vs. 100.0 cm, *p* = 0.001) and visceral fat (67.4 vs. 56.3 mm, *p* = 0.003), and were more frequently men (63.0% vs. 40.7%, *p* = 0.007), centrally obese (IDF 96.6% vs. 78.0%, *p* < 0.001) and dyslipidaemic (88.5% vs. 74.1%, *p* = 0.019). Notably, neither HbA1c (6.6% [49 mmol/mol] vs. 7.0% [53 mmol/mol], *p* = 0.784), fasting glucose nor diabetes duration differed between groups, and liver stiffness was identical (4.4 kPa in both).

In multivariable logistic regression (Table 3), male sex and waist circumference were independently associated with MASLD. In the waist-based model (Model A), the odds of MASLD were more than doubled in men (OR 2.49, 95% CI 1.26–4.94) and rose with waist circumference (OR 1.04 per cm, 95% CI 1.01–1.07), whereas age was not associated (OR 1.00, 95% CI 0.97–1.02). Because waist circumference and BMI were collinear and were therefore not entered jointly, an alternative BMI-based model (Model B) was fitted and confirmed the independent contribution of male sex (OR 2.71, 95% CI 1.43–5.17) and BMI (OR 1.07 per unit, 95% CI 1.01–1.13). For severe steatosis (S3), the Firth penalised model identified male sex (OR 3.34, 95% CI 1.03–10.76) and waist circumference (OR 1.07 per cm, 95% CI 1.03–1.11) as the independent factors associated with the outcome, with no association for age (based on 21 events; estimates should be interpreted cautiously).

### Graded relationship between waist circumference and hepatic phenotype

To characterise the relationship between abdominal adiposity and the hepatic phenotype, waist circumference was divided into quartiles (Q1 ≤ 98, Q2 > 98–107, Q3 > 107–116, Q4 > 116 cm) and related to MASLD, severe steatosis and fibrosis (Fig. 1; Table 4). The prevalence of MASLD increased monotonically across quartiles, from 55.8% in Q1 to 79.2%, 82.4% and 83.3% in Q2–Q4 (Cochran–Armitage test for trend *p* = 0.001). Severe steatosis showed a parallel gradient, rising from 0% in the lowest quartile to 8.3%, 9.8% and 25.0% in the highest (*p* < 0.001). In contrast, the prevalence of fibrosis (F ≥ 1) did not vary systematically across quartiles (17.3%, 18.8%, 21.6% and 12.5%; *p* = 0.65), indicating that the graded association with abdominal adiposity was specific to hepatic fat accumulation and was not mirrored by fibrosis.

**Fig. 2:**
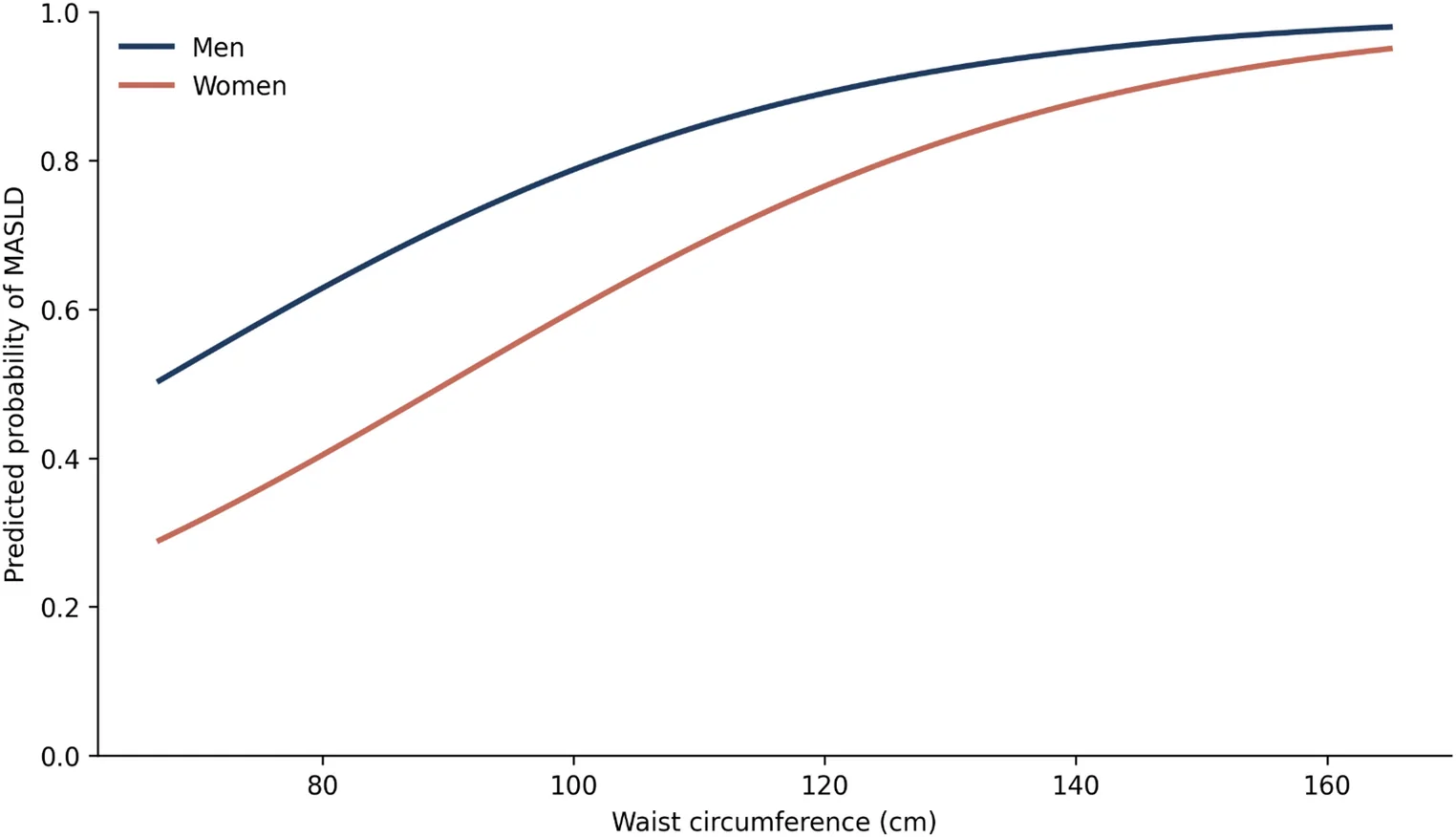
Independent contributions of sex and abdominal adiposity to MASLD. Model-based probability of MASLD across waist circumference for men and women, estimated from a logistic model including sex, age and waist circumference (age fixed at the cohort median). The probability rises with waist circumference in both sexes and the two curves remain approximately parallel, men lying on a uniformly higher trajectory; the sex–by–waist interaction was not significant (*p* = 0.36), indicating that male sex and abdominal adiposity are independently associated with MASLD

After adjustment for sex and age, the odds of MASLD relative to the lowest quartile rose across the distribution and reached significance from the third quartile onward (Q2 OR 2.45, 95% CI 0.97–6.18; Q3 OR 3.43, 95% CI 1.33–8.82; Q4 OR 3.24, 95% CI 1.24–8.45), with the adjusted risk plateauing in the upper half of the distribution. Consistent with this graded relationship, the sex-specific probability of MASLD increased progressively with waist circumference in both sexes, the curves remaining approximately parallel and offset, with men at a uniformly higher probability across the observed range and no statistical evidence that the effect of waist circumference differed by sex (sex–by–waist interaction *p* = 0.36) (Fig. 2). Male sex and waist circumference were thus independently associated with MASLD, with no evidence of a sex-by-waist interaction, so that the combination of male sex and greater abdominal adiposity defined the phenotype with the highest probability of MASLD.

### Exploratory analysis: glucose-lowering therapy and fibrosis

As a pre-specified secondary analysis, the association between therapy classes and the three hepatic outcomes was examined in separate models adjusted for age, sex and waist circumference, with Firth penalisation for the sparse outcomes (Supplementary Table S1). No therapy class was associated with the presence of MASLD or with severe steatosis, with the exception of a positive association between statin use and MASLD (OR 2.16, 95% CI 1.04–4.47). The signal was confined to prevalent fibrosis (F ≥ 1): GLP1-RA use was associated with a lower prevalence of fibrosis (OR 0.31, 95% CI 0.14–0.68) and SGLT2i use showed a concordant association (OR 0.38, 95% CI 0.15–0.93), whereas metformin showed a non-significant trend in the opposite direction (OR 2.06, 95% CI 0.92–4.59). These cross-sectional associations are hypothesis-generating and are interpreted with caution below.

## Discussion

In a real-world cohort of 219 outpatients with type 2 diabetes, three-quarters met criteria for MASLD and one in eight had severe steatosis, whereas clinically relevant fibrosis affected fewer than one in ten. Against this descriptive backdrop, male sex and abdominal adiposity—rather than age, glycaemic control or diabetes duration—were the factors independently associated with MASLD. That these adiposity- and sex-related factors retain their discriminating value in a population in which diabetes is universal is, in our view, the principal clinical message: the diagnosis of diabetes alone does not stratify the diabetic liver.

Our MASLD prevalence sits at the upper end of the published range. A recent meta-analysis of 123 studies and more than two million patients estimated the global pooled prevalence of NAFLD/MASLD in type 2 diabetes at approximately 65%, rising to nearly 69% in the most recent decade and reaching as high as 70% in the subgroup of diabetic patients with hypertension, hyperlipidaemia or the full metabolic syndrome [5]. Our figure of 75.3% is consistent with, and marginally above, this metabolically loaded subgroup, as expected for consecutively enrolled internal-medicine outpatients with established cardiometabolic disease. It also exceeds the 68.3% we previously reported in a broader, non-diabetes-restricted metabolic cohort from the same setting [9], in line with the recognised role of diabetes as a dominant driver of hepatic steatosis.

The fibrotic burden, by contrast, was lower than pooled estimates and merits frank discussion. In the same meta-analysis, the pooled prevalence of significant fibrosis (F ≥ 2) in type 2 diabetes was around 24.6% and advanced fibrosis (F ≥ 3) around 11.8% by transient elastography, rising to roughly 28% and 19% respectively among diabetic patients who already had MASLD [5]. Our corresponding figures (F ≥ 2 7.3%, F ≥ 3 5.5%) are substantially lower. Several non-exclusive explanations apply. First, the setting differs: our patients were consecutively enrolled internal-medicine outpatients rather than a hepatology-referred or biopsy-selected population, the latter being acknowledged to overestimate fibrosis. Second, the methods differ: we used two-dimensional shear-wave elastography with kPa thresholds that are not directly interchangeable with the transient-elastography cut-offs underpinning the pooled estimates. Third, glycaemic control in our cohort was relatively good (median HbA1c 6.6%, 49 mmol/mol), and metabolic compensation may be reflected in a less advanced fibrotic stage. Finally, shear-wave measurements are subject to signal attenuation at high BMI, which could lead to under-detection of fibrosis in a centrally obese population. The dissociation between a high steatotic burden and a contained fibrotic burden is nonetheless biologically coherent with the natural history of MASLD, in which steatosis is near-ubiquitous but only a subset progresses to fibrosis.

The strong, independent association of MASLD with male sex and waist circumference reproduces—and, in this diabetes-restricted cohort, amplifies—the pattern we previously reported in a mixed metabolic population [9] and that is well established in the literature. Men generally exhibit a higher prevalence and severity of steatotic liver disease, attributed to a more visceral pattern of fat distribution and to sex-hormone influences on hepatic lipid metabolism [10, 11], with convergence of risk in women after menopause as oestrogenic protection is lost [12, 13]. We deliberately modelled adiposity using waist circumference, a continuous proxy of central and visceral adiposity, rather than a dichotomous central-obesity category, both to avoid the data separation that dichotomisation induced and because the continuous measure is mechanistically more informative. Its independent contribution aligns with the concept, central to our pathophysiological framework [3], that dysfunctional visceral adipose tissue is an early driver of MASLD through increased free fatty acid flux and hepatic lipid loading [22, 23]. The finding that severe steatosis was independently associated with male sex and waist circumference, but not with age, reinforces the view that the drivers of fat accumulation and of fibrogenesis are partly distinct, and that steatosis severity should not be used as a surrogate for fibrosis risk. The graded increase in the prevalence and adjusted odds of MASLD across waist circumference quartiles strengthens this interpretation by demonstrating a biological gradient rather than a simple threshold effect, consistent with a continuous contribution of visceral adipose tissue to hepatic lipid loading. The near-parallel, vertically offset probability curves for men and women (Fig. 2), together with the non-significant sex–by–waist interaction, indicate that male sex and abdominal adiposity are independently associated with MASLD, so that the centrally obese man represents the phenotype with the highest probability of MASLD.

The most informative aspect of the analysis is its specificity (Fig. 1). The same waist circumference gradient that tracked the prevalence of MASLD also tracked the prevalence of severe steatosis, rising steeply across quartiles, yet it had no counterpart for fibrosis, which remained low and showed no systematic relationship with abdominal adiposity. This dissociation indicates that, within an already diabetic population, the factors underlying hepatic fat accumulation and those underlying fibrogenesis are only partially overlapping: abdominal adiposity is closely coupled to the quantity of intrahepatic fat, but the transition to fibrosis is governed by additional factors—genetic susceptibility, the duration and intensity of lipotoxic and inflammatory injury, and individual fibrogenic propensity—that are not captured by anthropometry.

These observations have direct implications for hepatic phenotyping in type 2 diabetes. They support a two-step conceptualisation in which sex and abdominal adiposity identify the diabetic patient most likely to harbour hepatic steatosis, while the assessment of fibrosis—the principal factor associated with liver-related and cardiovascular prognosis—requires dedicated tools rather than anthropometric proxies. In practice, waist circumference and sex are well suited to flagging steatosis and to prioritising metabolic intervention, but should not be relied upon to estimate fibrosis risk; a patient with modest abdominal adiposity is not thereby reassured against significant fibrosis, and conversely the centrally obese man, although very likely to have steatosis, is not by virtue of anthropometry alone identifiable as fibrotic. This argues in favour of sequential, adiposity-informed phenotyping in which case-finding for steatosis and staging of fibrosis are addressed by distinct, appropriately calibrated instruments, without implying causality or any change to current guideline recommendations.

A particularly informative negative finding is the absence of any difference in HbA1c, fasting glucose or diabetes duration between patients with and without MASLD. This indicates that, once diabetes is present, it is the adiposity phenotype rather than the degree or duration of hyperglycaemia that determines the steatotic liver, and it argues for hepatic risk stratification in diabetes that looks beyond glycaemic metrics to sex and central adiposity.

Our secondary, exploratory analysis suggested that the association of therapy with the hepatic phenotype was confined to fibrosis: GLP1-RA use was associated with a lower prevalence of measured fibrosis (OR 0.31, 95% CI 0.14–0.68) and SGLT2i use showed a concordant association (OR 0.38, 95% CI 0.15–0.93), whereas no class was associated with the presence of MASLD or with severe steatosis (Supplementary Table S1). This pattern is biologically plausible in light of recent randomised evidence: in the phase 3 ESSENCE trial of 1,197 patients with biopsy-defined MASH and stage 2–3 fibrosis (planned interim analysis in the first 800 randomised), semaglutide 2.4 mg achieved resolution of steatohepatitis without worsening of fibrosis in 62.9% of patients versus 34.3% on placebo, and improvement in liver fibrosis without worsening of steatohepatitis in 36.8% versus 22.4%, leading to regulatory approval of semaglutide for moderate-to-advanced MASH fibrosis in 2025 [24]. More broadly, the advent of agents capable of reducing hepatic steatosis—incretin-based and dual GIP/GLP-1 receptor agonists such as tirzepatide—makes accurate hepatic phenotyping in diabetes increasingly relevant, although our own cross-sectional observations remain strictly hypothesis-generating. Our cross-sectional associations cannot, however, be read as evidence of benefit. The temporal relationship between therapy and hepatic phenotype cannot be established; the drug classes overlap and the analysis is exposed to confounding by indication of uncertain direction and to reverse causation, as illustrated by the positive statin–MASLD association, which most plausibly reflects preferential prescribing rather than harm; the fibrosis outcome was sparsely populated and dominated by borderline F1 stiffness; and elastographic measurement is itself limited at high BMI. We therefore present these associations strictly as hypothesis-generating, to be tested in prospective designs, and draw no conclusion regarding causation.

## Study limitations

This study has limitations. It is single-centre, with a limited sample size, and is not representative of the general diabetic population; external validation in larger, multicentre cohorts is warranted, and the high prevalence observed in this all-T2DM cohort should not be extrapolated to the general non-diabetic population, in which MASLD prevalence is substantially lower. Several laboratory variables had substantial missingness and were confined to descriptive use and excluded from the regression models. Ultrasonographic assessment of steatosis is operator-dependent, although double, blinded reporting with excellent inter-operator agreement mitigated this, and elastographic fibrosis staging may be affected by adiposity and may underestimate the true fibrotic burden; B-mode ultrasonography may also miss mild steatosis, so the observed prevalence may, if anything, be an underestimate. Ultrasonographic visceral-fat thickness, although associated with MASLD univariably, was not entered into the multivariable models because of its collinearity with waist circumference and BMI. The cross-sectional design precludes causal inference for all reported associations. Its strengths include a real-world, consecutively enrolled diabetic cohort, comprehensive cardiometabolic and elastographic phenotyping, the use of penalised regression to obtain stable estimates where data separation would otherwise have been uninterpretable, and the explicit benchmarking of findings against contemporary pooled estimates.

## Conclusions

In conclusion, MASLD is highly prevalent and frequently severe in real-world type 2 diabetes, while clinically relevant fibrosis affects a minority and may be underestimated by elastography in this population. Within this already-diabetic cohort, male sex and central adiposity—not age or glycaemic control—continue to discriminate MASLD, and they do so in a graded manner: the prevalence and adjusted odds of MASLD, and the prevalence of severe steatosis, rise progressively with waist circumference. This gradient has no counterpart for fibrosis, indicating that the factors underlying hepatic fat accumulation and fibrogenesis are only partially overlapping. Hepatic phenotyping in diabetes should therefore be adiposity- and sex-informed and should not stop at the diagnosis of diabetes itself, while recognising that anthropometric markers identify steatosis risk but not fibrosis risk, which requires dedicated assessment.

**Table 1:** Baseline characteristics of the type 2 diabetes cohort (*N* = 219)

Variable	Median (IQR) / *n* (%)
Age, years	65.0 (57.5–73.0)
Diabetes duration, years	10.0 (3.0–18.0)
BMI, kg/m²	29.7 (26.4–33.4)
Waist circumference, cm	107.3 (98.0–116.0)
Visceral fat, mm	66.0 (50.1–78.4)
HbA1c, %	6.6 (6.1–7.8)
HbA1c, mmol/mol	49 (43–62)
Liver stiffness, kPa	4.4 (3.8–5.1)
Male sex	126 (57.5)
Central obesity (IDF)	183/199 (92.0)
Central obesity (NCEP-ATP III)	156/199 (78.4)
Metabolic syndrome (NCEP-ATP III)	117 (53.4)
Hypertension	172 (78.5)
Dyslipidaemia	186 (84.9)
Coronary artery disease	51 (23.3)
Statin	155 (70.8)
Metformin	127 (58.0)
GLP1-RA	115 (52.5)
SGLT2i	71 (32.4)
Insulin	34 (15.5)
Hepatic phenotype	
MASLD	165 (75.3)
Steatosis, any	165 (75.3)
S1 (mild)	66 (30.1)
S2 (moderate)	73 (33.3)
S3 (severe)	26 (11.9)
Fibrosis, F ≥ 1	38 (17.4)
Significant fibrosis, F ≥ 2	16 (7.3)
Advanced fibrosis, F ≥ 3	12 (5.5)

Data are median (interquartile range) or n (%). Waist circumference and the central-obesity definitions were available in 199 of 219 participants (90.9%); the corresponding percentages refer to this subgroup

**Table 2:** Univariate comparison of patients with and without MASLD

Variable	MASLD+ (*n* = 165)	MASLD− (*n* = 54)	*p*
Age, years	65.0 (59.0–73.0)	67.0 (56.2–73.8)	0.741
Diabetes duration, years	10.0 (3.0–18.0)	7.0 (1.0–15.0)	0.211
BMI, kg/m²	30.1 (26.8–34.1)	27.8 (22.9–32.6)	0.009
Waist circumference, cm	109.0 (100–118)	100.0 (90–112)	0.001
Visceral fat, mm	67.4 (53.8–81.2)	56.3 (40.3–71.5)	0.003
HbA1c, %	6.6 (6.2–7.8)	7.0 (5.9–7.9)	0.784
HbA1c, mmol/mol	49 (44–62)	53 (41–63)	0.784
Fasting glucose, mg/dL	111 (98–129)	115 (92–148)	0.824
Liver stiffness, kPa	4.4 (3.8–5.1)	4.4 (3.7–5.1)	0.845
Male sex, %	63.0	40.7	0.007
Central obesity (IDF), %	96.6	78.0	< 0.001
Metabolic syndrome, %	57.6	40.7	0.046
Dyslipidaemia, %	88.5	74.1	0.019
GLP1-RA, %	53.9	48.1	0.610
SGLT2i, %	32.9	31.5	0.956

Continuous variables are median (IQR); categorical variables are percentages. Central obesity refers to the 199 participants with measured waist circumference

**Table 3:** Multivariable logistic regression models for MASLD and for severe steatosis (S3)

Predictor	OR (95% CI)	*p*
Outcome: MASLD — Model A, waist-based (*n* = 199; 149 events)		
Male sex	2.49 (1.26–4.94)	0.009
Age	1.00 (0.97–1.02)	0.993
Waist circumference (per cm)	1.04 (1.01–1.07)	0.004
Outcome: MASLD — Model B, BMI-based (*n* = 219; 165 events)		
Male sex	2.71 (1.43–5.17)	0.002
Age	1.00 (0.98–1.03)	0.709
BMI (per unit)	1.07 (1.01–1.13)	0.016
Outcome: Severe steatosis (S3) — Firth penalised (*n* = 199; 21 events)		
Male sex	3.34 (1.03–10.76)	0.044
Age	0.99 (0.95–1.03)	0.541
Waist circumference (per cm)	1.07 (1.03–1.11)	< 0.001

Firth penalised likelihood was used where data separation occurred

**Table 4:** Hepatic phenotype across quartiles of waist circumference (*N* = 199)

Outcome / model	Q1 (≤ 98 cm)	Q2 (> 98–107)	Q3 (> 107–116)	Q4 (> 116)
Prevalence, *n*/*N* (%)				
MASLD	29/52 (55.8)	38/48 (79.2)	42/51 (82.4)	40/48 (83.3)
Severe steatosis (S3)	0/52 (0.0)	4/48 (8.3)	5/51 (9.8)	12/48 (25.0)
Fibrosis (F ≥ 1)	9/52 (17.3)	9/48 (18.8)	11/51 (21.6)	6/48 (12.5)
Adjusted OR for MASLD (95% CI)†				
vs. Q1 (reference)	1.00	2.45 (0.97–6.18)	3.43 (1.33–8.82)	3.24 (1.24–8.45)

Prevalence is shown as n/N (%); odds ratios for MASLD use the lowest quartile as reference († adjusted for sex and age). Trends across quartiles (Cochran–Armitage): MASLD *p* = 0.001, severe steatosis *p* < 0.001, fibrosis *p* = 0.65

## Supplementary Information

Below is the link to the electronic supplementary material.

Supplementary Material 1
