# Supplementary material for: MASLD in type 2 diabetes: steatosis–fibrosis burden and metabolic determinants in a real-world cohort
Source: Acta Diabetol. 2026 Sep 10;63(12):2157–66. doi: 10.1007/s00592-026-02791-5 (PMC13769719; doi:10.1007/s00592-026-02791-5)
Supplement: Supplementary file 1 [file 592_2026_2791_MOESM1_ESM.docx]

**Supplementary Material**

*MASLD in type 2 diabetes: steatosis–fibrosis burden and metabolic determinants in a real-world cohort*

**Supplementary Table S1.** *Exploratory associations between therapy classes and hepatic outcomes. Values are odds ratios (95% CI), each adjusted for age, sex and waist circumference; Firth penalised likelihood was used for severe steatosis and fibrosis. The DPP4i class was excluded (n = 3). No adjustment for multiplicity was applied across the 18 exploratory comparisons. *p < 0.05.*

| **Therapy** | **n exposed** | **MASLD** | **Severe steatosis (S3)** | **Fibrosis (F≥1)** |
| --- | --- | --- | --- | --- |
| Statin | 155 | 2.16 (1.04–4.47)* | 1.02 (0.34–3.04) | 0.59 (0.26–1.32) |
| Ezetimibe | 40 | 1.05 (0.45–2.44) | 0.91 (0.25–3.27) | 1.64 (0.69–3.93) |
| Metformin | 127 | 1.70 (0.85–3.39) | 1.93 (0.68–5.53) | 2.06 (0.92–4.59) |
| GLP1-RA | 115 | 1.20 (0.60–2.38) | 0.68 (0.26–1.81) | 0.31 (0.14–0.68)* |
| SGLT2i | 71 | 0.98 (0.47–2.06) | 0.83 (0.30–2.33) | 0.38 (0.15–0.93)* |
| Insulin | 34 | 0.82 (0.34–2.02) | 1.55 (0.46–5.26) | 0.91 (0.34–2.41) |
